# Supplementary material for: Chromosome-level assembly of the water buffalo genome surpasses human and goat genomes in sequence contiguity
Source: Nat Commun. 2019 Jan 16;10:260. doi: 10.1038/s41467-018-08260-0 (PMC6335429; doi:10.1038/s41467-018-08260-0)
Supplement: Supplementary file 1 — Supplementary Information [file 41467_2018_8260_MOESM1_ESM.pdf]

Supplementary Figures

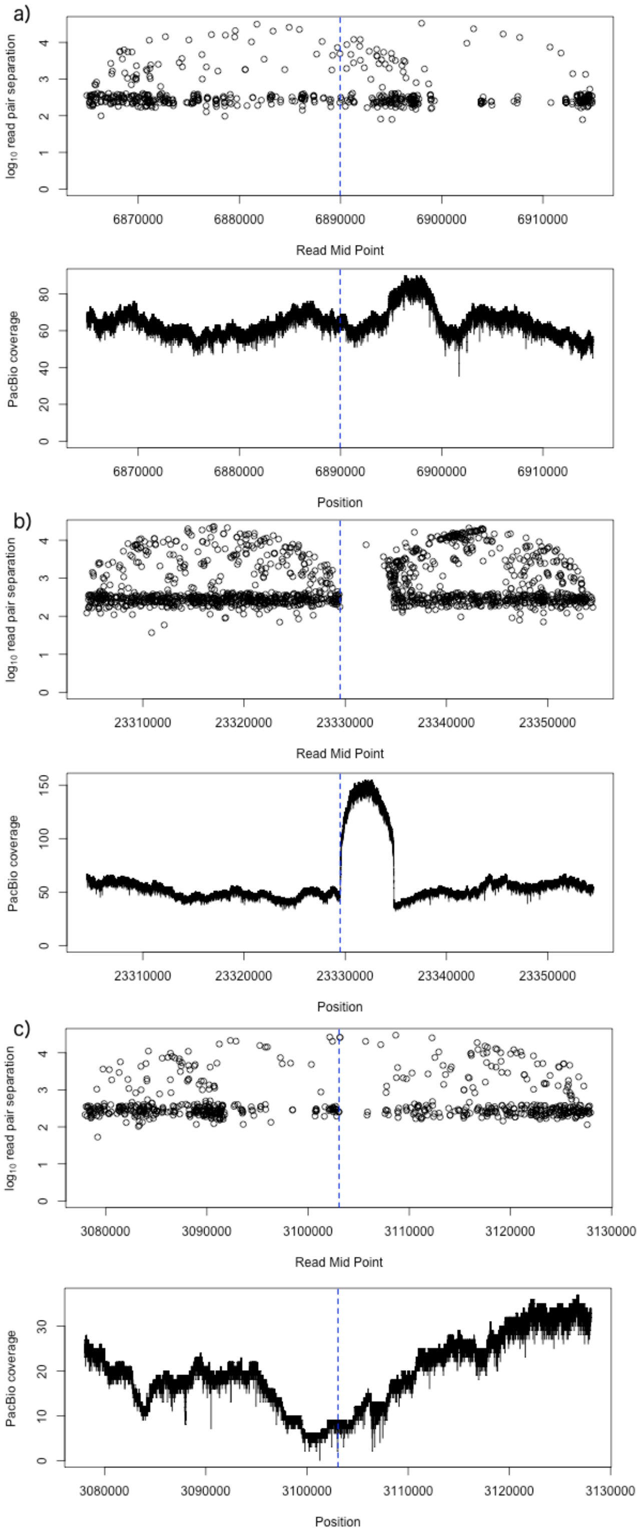

Supplementary Figure 1: **Examples of breakpoints introduced by HiRise**. Within each pair of panels, the top panel is log_{10} read pair separation of the aligned Chicago reads and the bottom panel is the PacBio coverage for the same region. Breakpoint is the dashed blue line. a) Breakpoint introduced in region with where the PacBio read coverage appears normal, b) Breakpoint in region with unusually high PacBio read coverage and c) Breakpoint in low PacBio coverage region.

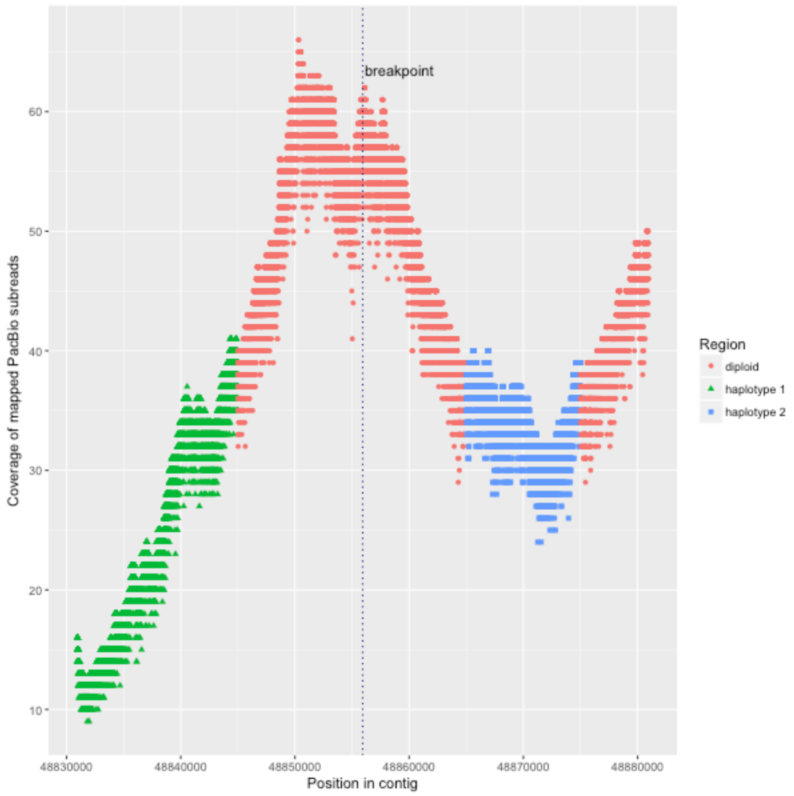

Supplementary Figure 2: **An illustration of phase shift around a breakpoint identified by HiRise.**

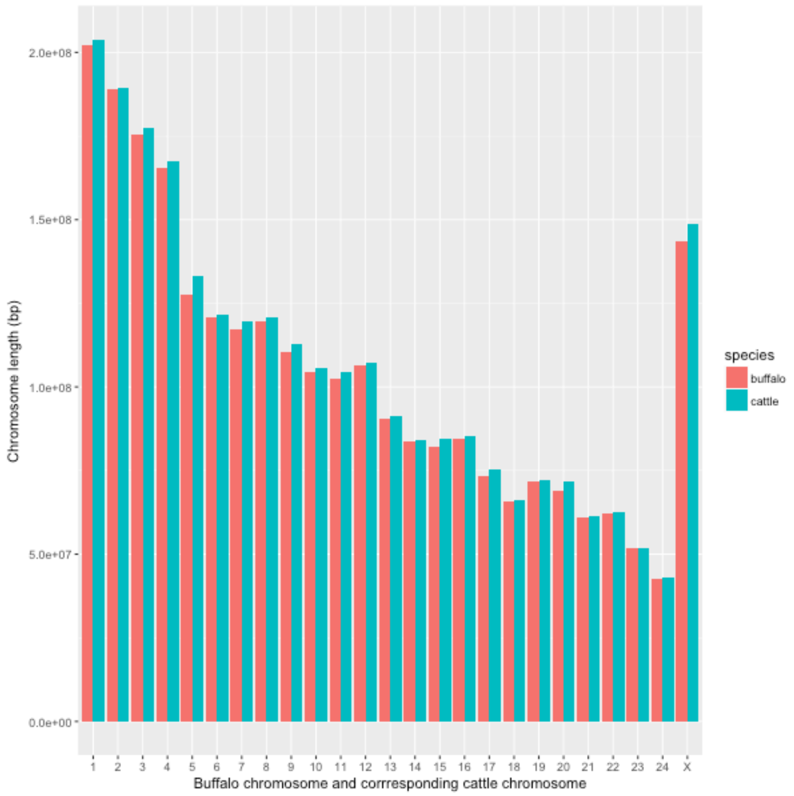

Supplementary Figure 3: **Chromosome length comparisons of water buffalo (UOA_WB_1) with cow (UMD 3.1).**

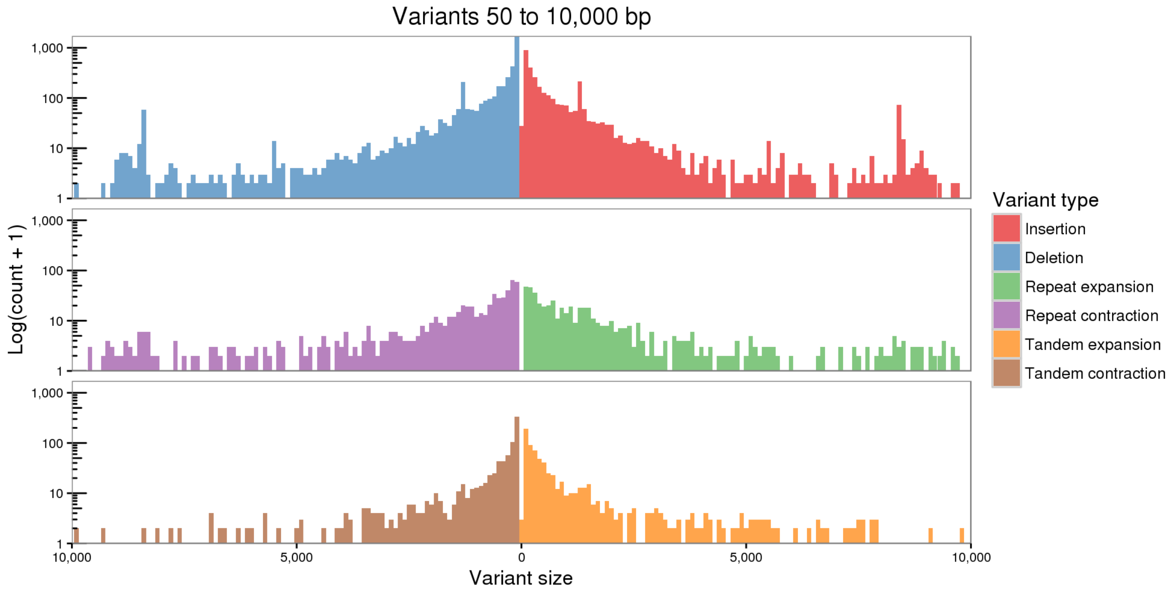

Supplementary Figure 4: **Distribution of structural variants when comparing haplotigs with the reference UOA_WB_1.**

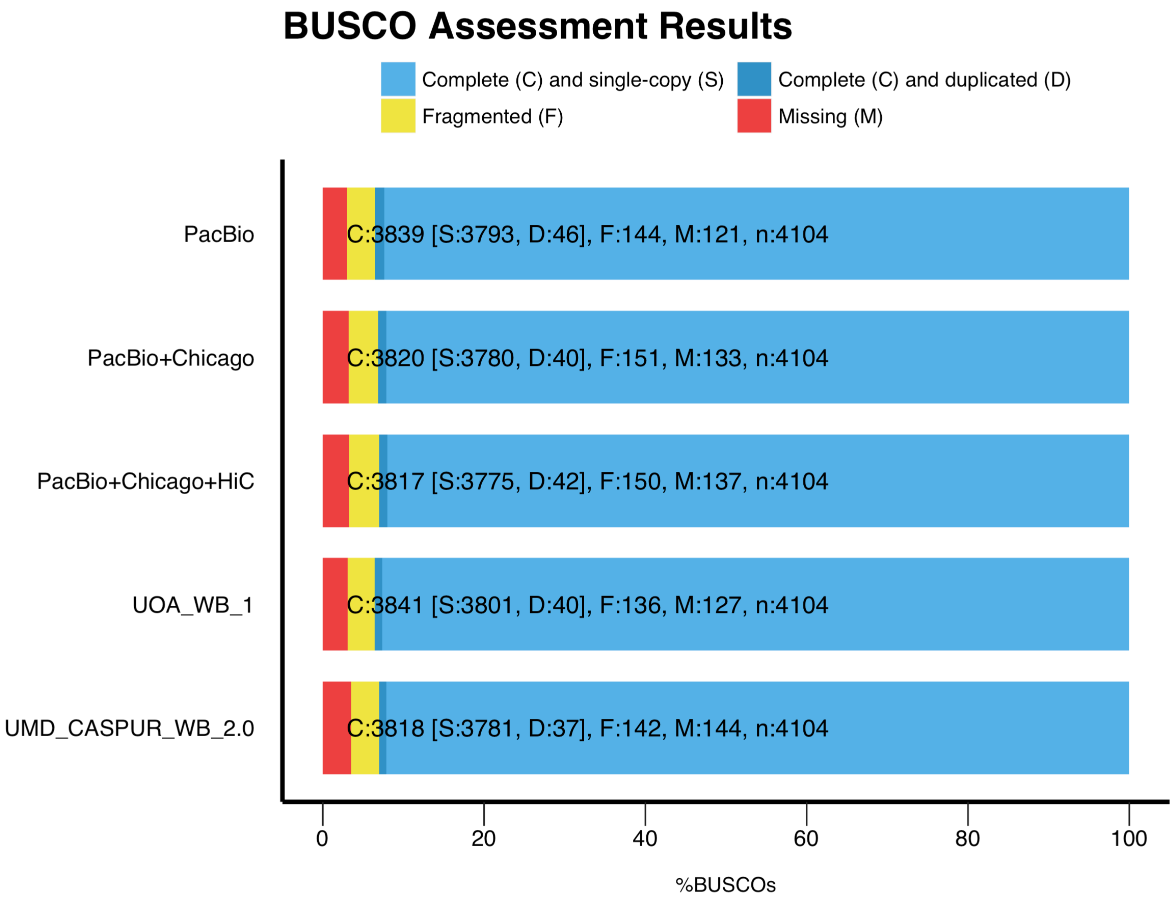

Supplementary Figure 5: **BUSCO assessments of the serial assembly stages.** The results of BUSCO assignments are given for assembly stages from initial PacBio based contigs to final chromosome level scaffolds. A comparison is also made to the previous short-read based buffalo genome (UMD_CASPUR_WB_2.0).

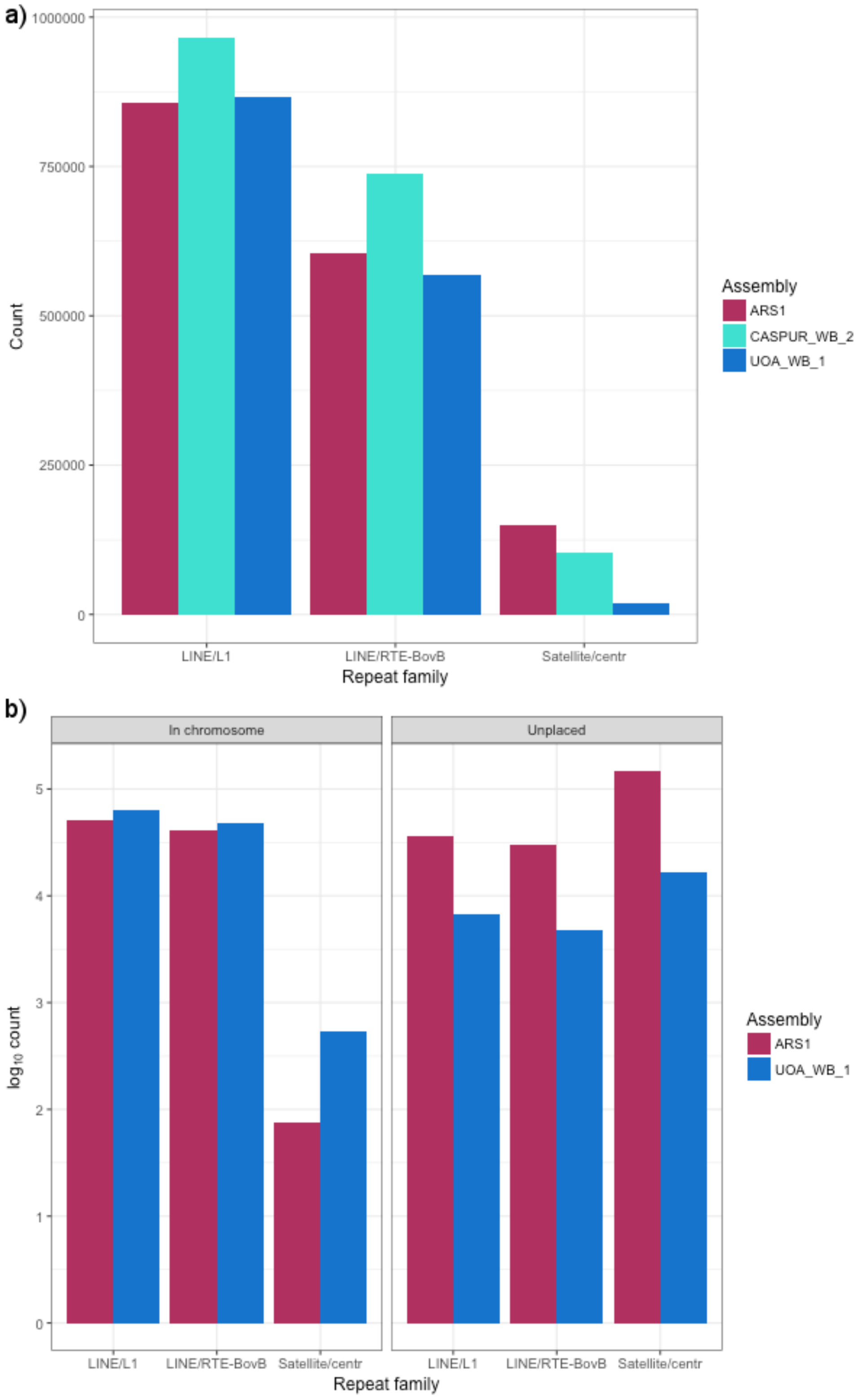

Supplementary Figure 6: **Three repeats families, LINE/L1, LINE/RTE-BovB and Satellite/centromeric, comparisons among assemblies.** a) Count of repeats in ARS1, UMD_CASPUR_WB_2.0 and UOA_WB_1 assemblies. b) log_{10} count of repeats in chromosome versus those in unplaced scaffolds between goat ARS1 and water buffalo UOA_WB_1.

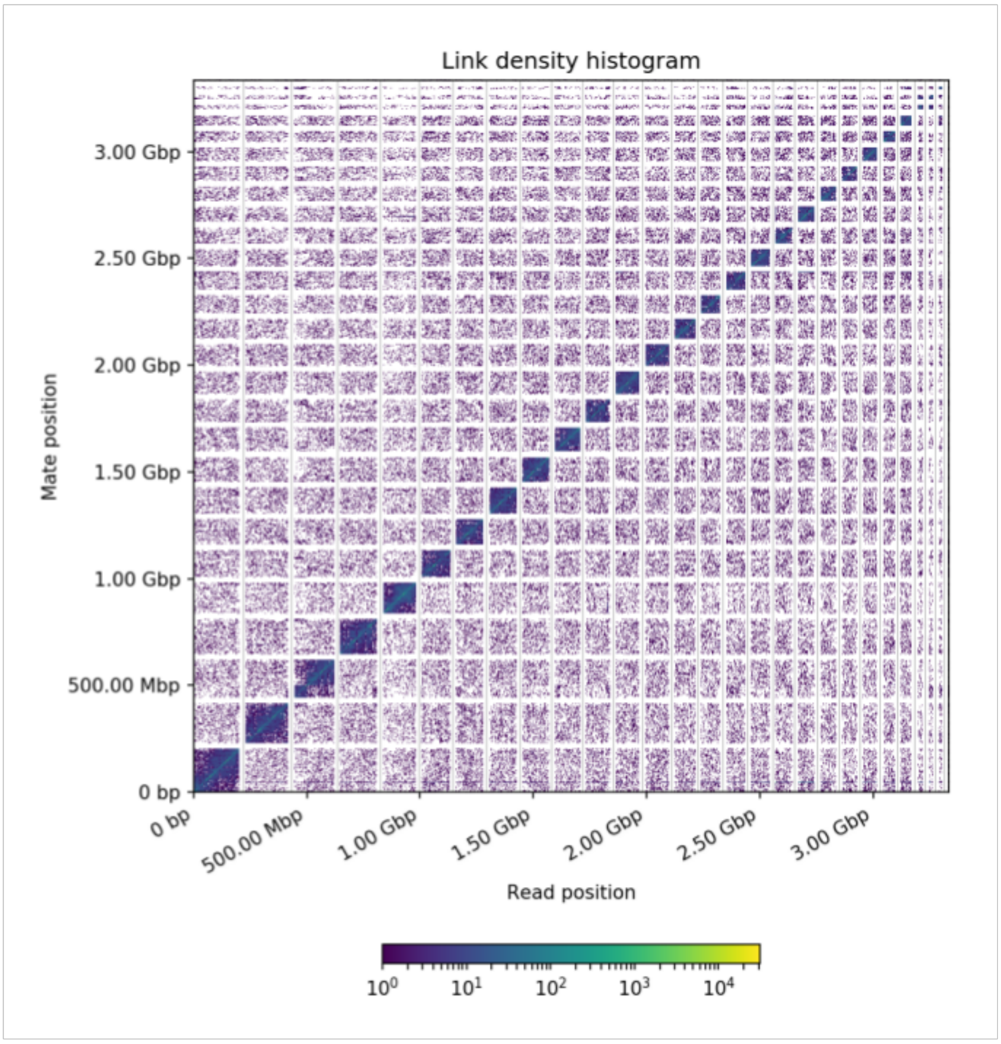

Supplementary Figure 7: **Clustering of scaffolds based on Hi-C reads.** The x- and y-axis represents the mapping positions of the first and second read in the read pair, respectively. The read pairs are assigned to bins and the colour of each square shows the number of read pairs within its bin. Scaffolds less than 1 Mb are excluded.

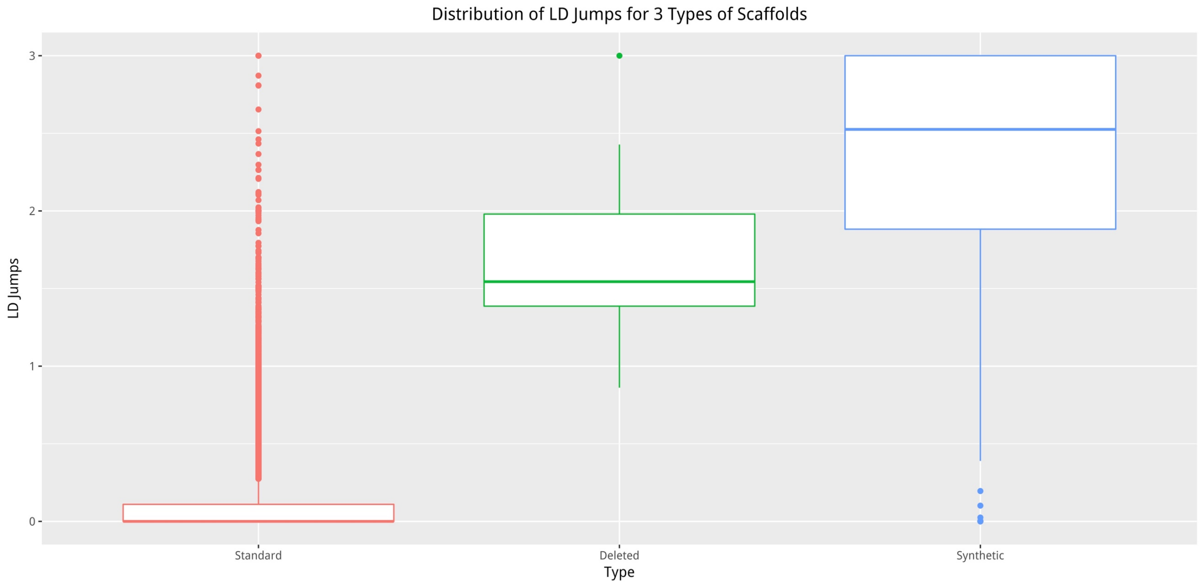

Supplementary Figure 8: **Distribution of LD jumps for three types of scaffolds.** LD jump is defined as the LDU difference of two consecutive SNPs along the sequence. ‘Standard’ refers to the LDU difference between all SNPs on the major scaffolds that carry sufficient SNPs to calculate LDU. ‘Deleted’ refers to LDU difference between SNPs that flank a series of 10Mb deletions at 10Mb intervals along longest scaffold, to simulate the joining of 2 discontiguous contigs. ‘Synthetic’ refers to the LDU for SNPs flanking every possible combination of scaffold pairs in all orientations, to detect scaffolds that are potentially neighbors. The outlier value of 0.275 was calculated from the distribution of standard LD jumps, defined as above the sum of 75th quantile and 1.5 times inter quartile range.

Supplementary Tables

Supplementary Table 1: **Alignments of buffalo (UOA_WB_1) chromosomes to cattle (UMD3.1) chromosomes.** The alignment was carried out with mashmap[1] v2 and filtered for sequences with more than 80% identity. There are five sub-metacentric buffalo chromosomes (i.e. chromosome 1 to 5) that each is homologous to two cattle chromosomes joined at a centromere. Due to indels, the proportion of aligned sequences can exceed 100%.

Buffalo chromosome	Cattle chromosome	Proportion of buffalo aligned in cattle (%)	Proportion of cattle aligned in buffalo (%)
1	1	77.5	99.3
1	27	22	99.2
2	23	27.7	100.3
2	2	72.2	99.8
3	19	36	99.2
3	8	63.4	98.9
4	5	72.4	99.4
4	28	27.6	99.2
5	29	40.6	101.1
5	16	60.2	94.3
6	3	100	99.7
7	6	99.9	99
8	4	99.9	99.4
9	7	99.9	98
10	9	99.3	99.1
11	10	101.2	99.6
12	11	100.1	99.6
13	12	100.4	100.4
14	13	99.9	99.4
15	14	100.3	97.9
16	15	99.7	99.3
17	17	100	98
18	18	100.2	100.8
19	20	100	99.9
20	21	99.7	96.4
21	22	99.9	99.3
22	24	99.9	99.1
23	26	99	99.6
24	25	100	99.4
X	X	100.4	99.6

Supplementary Table 2: **Assembly quality score values.**

Statistic	Description	UMD_CASPUR_WB_2.0	UOA_WB_1
QV	Quality value	36.46	41.96
COMPR_PE	Low CE-statistics computed on PE reads	141414	110744
HIGH_COV_PE	High read coverage areas	60344	3816
HIGH_NORM_COV_PE	High paired-read coverage areas	51907	3081
HIGH_OUTIE_PE	High number of mis-oriented or too distant PE reads	1150	36
HIGH_SINGLE_PE	High number of PE reads with unmapped pair	1710	28
HIGH_SPAN_PE	High number of PE reads with pair mapped in a different scaffold	191388	1304
LOW_COV_PE	Low read coverage areas	282257	37079
LOW_NORM_COV_PE	Low paired-read coverage areas	354658	38162
STRECH_PE	High CE-statistics computed on MP reads	148619	100124

Note: CE, compression/expansion; PE, paired-end

Supplementary Table 3: **Improvement of current assembly over previous short-read assembly.**

Description	UMD_CASPUR_WB_2.0	UOA_WB_1	Improvement
Total sequence length (bp)	2,836,166,969	2,655,780,776	
Total assembly gap length (bp)	74,388,041	373,500	
Number of contigs	630,368	953	
Contig N50 (bp)	21,938	22,441,509	+1023 fold
Contig L50	35,881	36	-997 fold
Number of scaffolds	366,983	509	
Scaffold N50 (bp)	1,412,388	117,219,835	+83 fold
Scaffold L50	581	9	-65 fold

Supplementary Table 4: **Genome annotation comparison between assemblies and species.**

Species	Protein coding genes	Partial CDS	Divergence time to buffalo (Myr)	RefSeq assembly accession	Annotation release ID

Bubalus bubalis	20,801	157	-	GCF_003121395.1	101
Bubalus bubalis	21,711	1,515	-	GCF_000471725.1	100
Bos Taurus	21,295	1,589	12.3	GCF_000003055.6	105
Capra hircus	20,755	457	24.6	GCF_001704415.1	102
Ovis aries	20,645	758	24.6	GCF_000298735.2	102
Sus scrofa	24,205	4,112	62	GCF_000003025.5	105
Homo sapiens	20,203	533	96	GCF_000001405.38	109

Supplementary Table 5: **Number and total base counts of insertions and deletion errors corrected by Pilon.**

**chromosome**	**no of insertion**	**insertion (bp)**	**no of deletion**	**deletion (bp)**
1	10708	14083	2753	12704
2	10967	14344	3275	14815
3	9442	12496	2834	12235
4	9980	13087	2964	13838
5	7978	10485	2442	11213
6	6921	9028	2086	9846
7	6491	8435	1692	7444
8	5303	6637	1001	4192
9	6653	8826	1942	9152
10	5683	7216	1432	6126
11	6175	8061	1615	7232
12	5235	6825	1481	6439

13	6490	8368	1872	8131
14	2749	3342	580	1727
15	4601	6058	1347	6858
16	5380	6989	1640	6989
17	4118	5453	1206	5131
18	3583	4568	1232	4662
19	3867	5048	1091	4922
20	4060	5363	1286	5433
21	3301	4316	1041	4912
22	3444	4508	1053	5452
23	2897	3802	892	3825
24	1961	2596	683	3051
X	7118	8999	1969	6890
**TOTAL**	**145105**	**188933**	**41409**	**183219**

Supplementary Note 1

Contig assembly

```
# FALCON CONFIGURATION
# FALCON version 0.7.0
# FALCON-Unzip git commit 7ebc99c4c9cf9770eec5399814402a33ecb73e65
[General]

# list of files of the initial subread fasta files
input_fofn = input.fofn

input_type = raw
#input_type = preads

# The length cutoff used for seed reads used for initial mapping
genome_size = 2900000000
#seed_coverage = 30
length_cutoff = 12000

# The length cutoff used for seed reads for pre-assembly
length_cutoff_pr = 12000

use_tmpdir = /scratch
job_queue = bigmem
sge_option_da = -pe smp 4
sge_option_la = -pe smp 20
sge_option_pda = -pe smp 6
sge_option_pla = -pe smp 16
sge_option_fc = -pe smp 24
sge_option_cns = -pe smp 8

# concurrency setting
default_concurrent_jobs = 384
pa_concurrent_jobs = 384
cns_concurrent_jobs = 384
ovlp_concurrent_jobs = 384
```

# overlapping options for Daligner
pa_HPCdaligner_option = -v -dal128 -e0.75 -M24 -l1200 -k14 -h256 -w8 -s100 -t16
ovlp_HPCdaligner_option = -v -dal128 -M24 -k24 -h600 -e.95 -l1800 -s100

pa_DBsplit_option = -x500 -s400
ovlp_DBsplit_option = -s400

# error correction consensus option
falcon_sense_option = --output_multi --min_idt 0.70 --min_cov 4 --max_n_read 200 --n_core 24

# overlap filtering options
overlap_filtering_setting = --max_diff 120 --max_cov 120 --min_cov 2 --n_core 12

Checks for contig joins

The code details of which chromosomal region gets reordered can be found at https://github.com/lloydlow/BuffaloAssemblyScripts

Gaps comparisons

Below are sample commands to generate gap positions and ungapped contigs for analysis using seqtk (https://github.com/lh3/seqtk).

#to get gap positions
seqtk cutN -n 3 -g /<PATH-TO-FILE>/gap_genome_analysis/species/human/human_chr_only.fa > /fast/users/a1223107/gap_genome_analysis/species/human/human_chr_only.coor

#to get ungapped contigs
seqtk cutN -n 3 /<PATH-TO-FILE>/gap_genome_analysis/species/human/human_chr_only.fa > /fast/users/a1223107/gap_genome_analysis/species/human/human_chr_only_ungapped.fa

The files generated from above commands were then analysed using R scripts (https://github.com/lloydlow/BuffaloAssemblyScripts).

Further assembly evaluation

We assessed the error rates of the previously published UMD_CASPUR_WB_2.0 water buffalo reference assembly and UOA_WB_1 assembly using alignments of Illumina short reads as previously described[2]. Short-insert Illumina WGS reads from the reference animal, Olimpia, were aligned to both assemblies using BWA MEM[3]. We used the reference-free assembly validation software, FRCBam[4] to generate feature response curves for both assemblies, and to identify compression/expansion (CE) errors in assembly sequence. We further identified candidate erroneous bases in each assembly using FreeBayes[5]. Following the methods previously used to benchmark the goat ARS1 reference assembly, we generated an assembly Quality Value (QV) for the UMD_CASPUR_WB_2.0 assembly and our assembly using the identified FreeBayes polymorphic sites. In order to distinguish between legitimate heterozygous sites and single nucleotide errors in the assemblies, we increased the threshold for FreeBayes polymorphic site calling (-F) from 0.5 to 0.75. Commands used to generate all assembly quality assessment metrics can be found in the GitHub repository (https://github.com/lloydlow/BuffaloAssemblyScripts).

Based on the short-read alignments from the reference individual to both assemblies, we found that UOA_WB_1 and UMD_CASPUR_WB_2.0 had QVs of 41.96 and 36.46, respectively. The 5-point QV difference between these two assemblies indicates that our new long-read reference assembly contains nearly half an order of magnitude fewer single nucleotide errors than in UMD_CASPUR_WB_2.0. This is despite the previously reported higher error rates for long-read-based reference genome assemblies. We also found a substantial reduction in the occurrence of discordant paired end reads (HIGH_OUTIE_PE, HIGH_SINGLE_PE and HIGH_SPAN_PE; see supplementary table 2), suggesting that we have corrected several misassembled regions in UMD_CASPUR_WB_2.0. Finally, we found at least a ten-fold reduction in high coverage regions (HIGH_COV_PE and HIGH_NORM_COV_PE) in UOA_WB_1, suggesting that we have eliminated a number of compressed repetitive regions found in UMD_CASPUR_WB_2.0.

References

1. Jain, C., Dilthey, A., Koren, S., Aluru, S. & Phillippy, A. M. A fast approximate algorithm for mapping long reads to large reference databases. *bioRxiv* 103812 (2017). doi:10.1101/103812
2. Bickhart, D. M. *et al.* Single-molecule sequencing and chromatin conformation capture enable de novo reference assembly of the domestic goat genome. *Nat. Genet.* **49,** 643–650 (2017).
3. Li, H. & Durbin, R. Fast and accurate short read alignment with Burrows--Wheeler transform. *Bioinformatics* **25,** 1754–1760 (2009).
4. Vezzi, F., Narzisi, G. & Mishra, B. Reevaluating Assembly Evaluations with Feature Response Curves: GAGE and Assemblathons. *PLoS One* **7,** e52210 (2012).
5. Garrison, E. & Marth, G. Haplotype-based variant detection from short-read sequencing. *arXiv:1207.3907* (2012).
